# Demographics and Infection Sources in Coding-Defined Sepsis at a Romanian County Emergency Hospital: A Retrospective Single-Center Study

**DOI:** 10.7759/cureus.117040

**Published:** 2026-09-27

**Authors:** Sulaymaan Ahmed, Muhammad Asim Mukhtar, Camer Salim

**Affiliations:** 1 General Medicine, Ovidius University of Constanța, Constanța, ROU; 2 Medicine, Ovidius University of Constanța, Constanţa, ROU; 3 Emergency Medicine, "Sf. Apostol Andrei" Emergency County Clinical Hospital, Constanța, ROU

**Keywords:** coding-defined sepsis, constanța, emergency department, hospital epidemiology, icd-10, infection source, sepsis, urinary tract infection

## Abstract

Background: Sepsis is one of the major causes of hospital morbidity and mortality, and its epidemiology varies with local patient populations. Descriptive data from Romanian county hospitals remain limited. We have described the demographic profile, infection sources, and hospital service distribution of patients with coding-defined sepsis at a Romanian county emergency hospital over a full calendar year.

Methods: This was a retrospective, single-center study at the "Sf. Apostol Andrei" Emergency County Clinical Hospital, Constanța, Romania. Using the hospital electronic discharge registry (Hipocrate OPIS Externari), discharge records were filtered by infectious-disease diagnostic category and individually reviewed by clinicians, yielding 1,147 records of patients presenting to the emergency department with infection-related diagnoses during the 2025 calendar year. The study included patients with sepsis as their primary diagnosis (International Classification of Diseases, Tenth Revision (ICD-10) A40-A41). We recorded and deidentified age, sex, discharge service, primary infection source, and length of stay. The analysis is primarily descriptive. We excluded toxic shock syndrome and other specified infections.

Results: Of 1,147 infection-related discharge records reviewed, 129 (11.2% of infection-related records; 0.39% of all 33,250 emergency department admissions during the study period) carried a principal discharge diagnosis of coding-defined sepsis (ICD-10 A40 or A41). The cohort was predominantly adult (115/129, 89.1%); among adults, the mean age was 60.2 years (standard deviation (SD), 16.7), while the combined mean age, including 14 pediatric patients, was 54.3 years (SD, 23.2). Of the 129 patients, 82 (63.6%) were male and 45 (34.9%) were female patients, and sex was indeterminate in two (1.6%). General surgery (36%) and urology (32%) were the most frequent discharge services. The leading infection source was urinary (41%), followed by abdominal (26%) and skin and soft tissue (16%). Median length of stay was seven days (interquartile range, 4-14 days; mean 10.0 days; n = 126 of 129).

Conclusions: Over a full calendar year at this Romanian county clinical emergency hospital, sepsis predominantly affected older patients managed by general surgery and urology, with urinary and abdominal sources dominating. These descriptive regional data add to the limited hospital-based descriptive evidence on sepsis in Romania.

## Introduction

Sepsis is defined by the Third International Consensus (Sepsis-3) as a life-threatening organ dysfunction caused by a dysregulated host response to infection [1]. Septic shock represents a more severe subset characterized by circulatory and cellular-metabolic failure, historically associated with in-hospital mortality exceeding 40% [1]. Sepsis may arise from a wide range of infectious sources, from community-acquired urinary and respiratory infections to postprocedural and healthcare-associated presentations.

Sepsis is among the leading causes of in-hospital mortality and a major driver of intensive care resource utilization worldwide. The Global Burden of Disease study estimated 48.9 million incident cases of sepsis in 2017, accounting for approximately 11 million deaths and representing nearly 20% of all global mortality in that year [2]; updated analyses confirm that the burden remains substantial with marked regional variation in incidence and outcome [3]. In the emergency department, sepsis represents a time-critical diagnostic and therapeutic challenge. The Surviving Sepsis Campaign guidelines recommend initiation of blood cultures, broad-spectrum antibiotics, and intravenous fluid resuscitation within the first hour of recognition, with vasopressor support commenced promptly when septic shock criteria are met [4]. The primary source of infection strongly influences the clinical trajectory; urinary, abdominal, and respiratory sources each carry distinct implications for empirical antimicrobial selection, the need for surgical source control, and expected outcome, and their relative distribution varies considerably between institutions according to case-mix, referral patterns, and local microbial epidemiology [3,5].

Compared with Western European and North American literature, published hospital-based sepsis epidemiology from county-level emergency hospitals in Romania remains relatively limited; to our knowledge, no study has previously described the demographic profile, infection sources, or hospital service distribution of coding-defined sepsis at Constanța County Emergency Hospital, which is located in the southeastern Black Sea coast region of Romania. The primary objective of this study was to describe the demographic characteristics, infection sources, discharge services, and length of hospital stay among patients with International Classification of Diseases, Tenth Revision (ICD-10), coding-defined sepsis admitted through the emergency department of a Romanian county clinical emergency hospital.

## Materials and methods

We performed a retrospective, single-center, observational study at the "Sf. Apostol Andrei" Emergency County Clinical Hospital, Constanța, a county emergency hospital in southeastern Romania. Data were obtained from the Hipocrate OPIS Externari (the hospital's electronic discharge registry, in which administrative and diagnostic data for all admitted patients are recorded and ICD-10 diagnostic coding is performed). Of 33,250 patients who presented to the emergency department and were admitted between January 1 and December 31, 2025, we filtered and reviewed discharge records in the Hipocrate OPIS Externari system to identify infection-related principal diagnoses. We selected a one-year study timeframe to capture seasonal variation in infection presentation patterns and provide sufficient case volume for descriptive analysis at a single institution. Two Emergency Department clinicians used the Hipocrate OPIS Externari system to filter discharge records by infectious-disease diagnostic category, identifying an initial set of records with infection-related and sepsis diagnoses; each returned record was then individually reviewed by the clinicians to confirm relevance and assign it to the appropriate diagnostic category, rather than being accepted without clinical verification. The reviewing clinicians had access to the complete electronic medical record within the Hipocrate system for case identification and source classification; only deidentified variables were subsequently provided to the investigator for analysis. A category-based filter followed by individual clinical review was used deliberately.

Reviewers applied a consistent categorical framework (infectious and parasitic diseases, respiratory, urinary, abdominal/surgical, and skin and soft-tissue infections); the sepsis analysis cohort was defined by a principal diagnosis of A40 or A41. Representative ICD-10 codes observed within each category included, but were not limited to: infectious and parasitic diseases (Chapter I, e.g., A49.9, A09, A15.0-A18.0, B67.0-B67.1); respiratory infections (e.g., J18.9, J02.9, J03.9); urinary infections (e.g., N39.0, N18.8, N20.1-N20.2); abdominal/surgical infections (e.g., K35.9, K61.0, K65.0, K80.00, K85); and skin and soft-tissue infections (e.g., L02.9). As category assignment was performed by clinician judgment within the Hipocrate system rather than a predefined, closed ICD-10 code list, this is a representative rather than exhaustive enumeration of the codes encountered. Discrepancies were resolved by consensus. As reviewers classified records jointly rather than independently, interrater reliability could not be quantified, and case ascertainment was not validated by a third party; both were acknowledged limitations of the study denominator. This category-based filtering and individual clinical review process yielded 1,148 infection-related records. One record was excluded because it did not correspond to a genuine patient discharge and appeared to be a nonpatient administrative test entry; this left a final denominator of 1,147 hospital inpatient discharge records for patients who presented to the emergency department with infection-related diagnoses. These records represent patients admitted through the emergency department and subsequently managed by different inpatient services across the institution. Identified records were extracted and compiled by the clinical reviewers directly from the Hipocrate OPIS Externari system and given as a printed register output; data were transcribed from this printed document for analysis. The participant flow from total admissions through to the sepsis cohort is shown in Figure 1. This study is reported in accordance with the STrengthening the Reporting of OBservational studies in Epidemiology (STROBE) guidelines for cohort studies; a completed STROBE checklist is provided in the Appendix [6].

**Figure 1:**
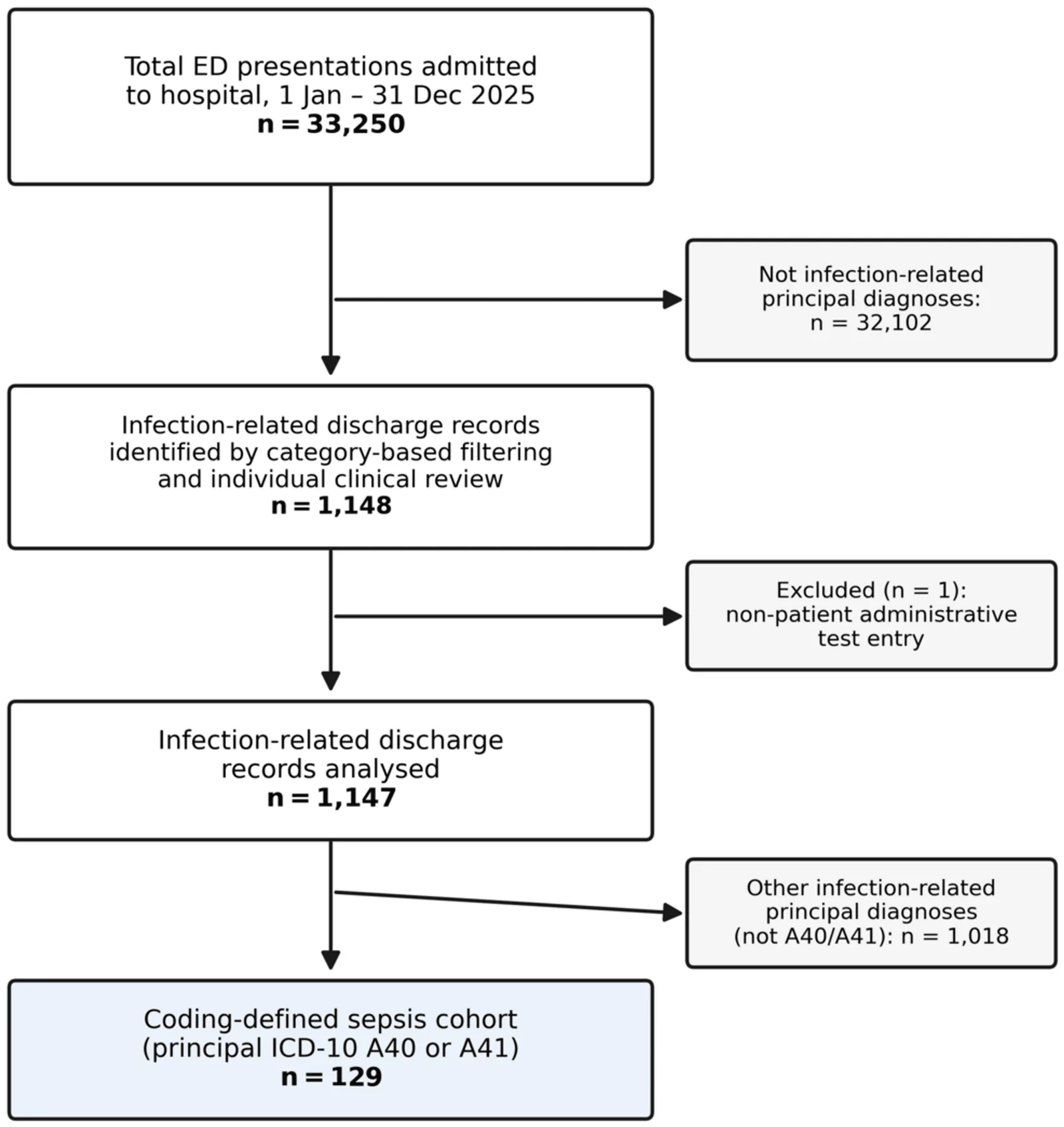
Participant flow diagram Of 33,250 ED presentations admitted to the hospital between January 1 and December 31, 2025, 1,148 infection-related discharge records were identified by category-based filtering of the discharge register, followed by individual clinical review; one nonpatient administrative test entry was excluded, giving 1,147 records for analysis. Within these, the coding-defined sepsis cohort was defined by a principal discharge diagnosis of ICD-10 A40 or A41 (n = 129) ED: emergency department; ICD-10: International Classification of Diseases, Tenth Revision

Patients of any age were included if their principal discharge diagnosis was sepsis, identified by ICD-10 codes A40 (streptococcal sepsis) or A41 (other and unspecified sepsis); throughout this report, the term "coding-defined sepsis" refers to patients identified by these codes, thus distinguishing this administrative definition from clinically adjudicated sepsis, which would require verification of organ dysfunction criteria. We classified patients under 18 years as pediatric and those 18 years and older as adults. Patients whose principal diagnosis was a localized or other specified infection, including toxic shock syndrome (A48.3), gas gangrene (A48.0), and erysipelas (A46), were excluded, so that the cohort comprised only patients coded specifically as having sepsis. ICD-10 codes A40 and A41 were selected as they represent the principal ICD-10 designation for sepsis as a primary condition; cases in which sepsis appeared as a secondary diagnosis under a different principal code, or in which bacteremia was recorded under organism-specific codes outside the A40-A41 range (such as Salmonella septicemia, A02.1), would not have been captured, and this approach may therefore underestimate the true institutional sepsis burden; the selection of A40 and A41 as identification codes is consistent with prior administrative sepsis studies utilizing principal diagnosis coding [7]. The registry coding did not contain a distinct identifier for septic shock, and no hemodynamic, vasopressor, or intensive care data were available; the severity stage (sepsis vs. septic shock), therefore, could not be determined and was not claimed.

For each included patient, we recorded age, sex, discharge service (the inpatient clinical service responsible for the patient's care and discharge), primary infection source, and length of stay. Sex and age were obtained from administrative registry data; direct identifiers were discarded, and only deidentified variables were used for analysis. The primary infection source was categorized as urinary, abdominal, skin and soft tissue, biliary, pulmonary, or unknown, based on the admission and principal diagnoses rather than on microbiological confirmation. Where a patient was discharged more than once with a sepsis diagnosis, only the first admission was counted, so that the cohort reflects unique patients. Where a single admission involved transfer between departments, the episode was counted once and attributed to the discharging service, so that each record represents one unique patient admission. Where multiple potential infection sources were identifiable from the discharge record, the source most closely linked to the principal discharge diagnosis was selected. Source assignment was performed by an emergency medicine clinician using the predefined diagnostic categories described above, without independent verification. Records containing principal diagnoses of pyelonephritis, urosepsis, or urinary tract infection were assigned to the urinary source category; those with peritonitis, perforated viscus, or intra-abdominal infection codes were assigned to the abdominal source category; and those with cellulitis, necrotizing fasciitis, or wound infection codes were assigned to the skin and soft-tissue category. Comorbidity, illness-severity, microbiological, and in-hospital outcome data were not recorded in the register and could not be analyzed.

A coding-based case definition was used because it enables consistent, reproducible identification of cases from routinely collected data and permits comparison with other administrative sepsis studies. No formal sample size calculation was performed; all eligible cases identified during the study period were included.

Continuous variables are summarized as mean (standard deviation (SD)) or median (interquartile range (IQR)), with range reported where informative, and categorical variables as counts and percentages. The analysis was primarily descriptive. As a single exploratory comparison, age was compared between the two most frequent infection-source groups (urinary and abdominal) using the Mann-Whitney U test; a nonparametric test was selected because Shapiro-Wilk testing indicated nonnormal age distribution in the urinary-source group (W = 0.931, p = 0.004) but not in the abdominal-source group (W = 0.961, p = 0.256). A two-sided p value <0.05 was considered statistically significant; as this was a single exploratory comparison with no adjustment for other variables, findings should be considered hypothesis-generating rather than confirmatory. Statistical analyses were performed using jamovi version 2.7.31 (the jamovi project, Sydney, Australia).

Ethics approval for access to and analysis of the institutional patient records used in this study was granted by the Ethics Committee for the Approval of Clinical Studies and Research of the Emergency County Clinical Hospital "Sf. Apostol Andrei" Constanța, Romania (approval no. 8449/06.02.2026). Given the retrospective design and the use of deidentified records, the requirement for individual informed consent was waived, and no patient-identifying information is reported.

## Results

Of 33,250 emergency department (ED) presentations admitted to the hospital during the full year 2025, 1,147 (3.4%) had infection-related principal discharge diagnoses; of these, 129 (11.2% of infection-related records, not of all ED admissions) had a principal discharge diagnosis of sepsis (ICD-10 A40 or A41) and formed the study cohort (Figure 1).

The mean age was 54.3 years (SD, 23.2; median, 59; range, 1-93). Fourteen patients were pediatric, and among the 115 adults, the mean age was 60.2 years (SD, 16.7). The cohort is predominantly adult; pediatric patients (n = 14, 10.9%) were retained rather than excluded to provide a complete institutional picture of all coding-defined sepsis cases across age groups during the study year. Given the fundamental differences in sepsis epidemiology between pediatric and adult populations, adult-specific statistics are provided separately, and the two groups are presented independently in Table 1; the adult population remains the primary focus of this study. Because the register did not permit separate clinical stratification by age group, combined-cohort summary statistics are influenced by the inclusion of younger patients, and readers are directed to the adult-specific figures in Table 1 where available. Eighty-two (63.6%) were male and 45 (34.9%) were female patients, and two (1.6%) had sex that could not be determined from administrative registry data, most likely owing to absent or unreadable identifier fields.

**Table 1:** Demographic and infection-source characteristics of adult and pediatric patients with coding-defined sepsis (n = 129). Infection source was assigned from discharge diagnosis codes and is not microbiologically confirmed SD: standard deviation

Characteristic	Adult (n = 115)	Pediatric (n = 14)	Total (n = 129)
Age, mean (SD), years	60.2 (16.7)	6.0 (4.6)	54.3 (23.2)
Age, median (range), years	63 (19-93)	6 (1-15)	59 (1-93)
Male sex, n (%)	72 (62.6%)	10 (71.4%)	82 (63.6%)
Female sex, n (%)	41 (35.7%)	4 (28.6%)	45 (34.9%)
Indeterminate sex, n (%)	2 (1.7%)	0 (0.0%)	2 (1.6%)
Infection source, n (%)			
Urinary	53 (46.1%)	0 (0.0%)	53 (41.1%)
Abdominal	31 (27.0%)	3 (21.4%)	34 (26.4%)
Skin and soft tissue	21 (18.3%)	0 (0.0%)	21 (16.3%)
Unknown	8 (7.0%)	10 (71.4%)	18 (14.0%)
Biliary	2 (1.7%)	0 (0.0%)	2 (1.6%)
Pulmonary	0 (0.0%)	1 (7.1%)	1 (0.8%)

Infection source distribution differed markedly between age groups: urinary-source sepsis was confined entirely to adult patients, whereas the source was unknown in 10 of 14 pediatric patients (71.4%), compared with eight of 115 adult patients (7.0%). The high proportion of unclassified infection sources in the pediatric group may relate to the more heterogeneous clinical presentations of sepsis in younger patients, in whom a single identifiable infection source may not be readily documentable from discharge coding alone. The characteristics of both groups are presented in Table 1.

Most patients were managed by general surgery (47 patients, 36%) and urology (41, 32%), followed by nephrology (16, 12%), pediatric surgery (eight, 6%), and internal medicine (five, 4%); the remaining 12 patients (9.3%) were managed across pediatrics (n = 4, 3.1%), neonatology (n = 2, 1.6%), gastroenterology (n = 1, 0.8%), and other inpatient services (n = 5, 3.9%). The full service distribution is presented in Table 2. Together, general surgery and urology accounted for approximately 68% of the cohort.

**Table 2:** Distribution of sepsis patients by admitting service (n = 129). General surgery and urology together accounted for 68.2% of admissions

Discharge service	Value, n (%)
General surgery	47 (36.4)
Urology	41 (31.8)
Nephrology	16 (12.4)
Paediatric surgery	8 (6.2)
Internal medicine	5 (3.9)
Pediatrics	4 (3.1)
Neonatology	2 (1.6)
Gastroenterology	1 (0.8)
Other inpatient services	5 (3.9)
Total	129 (100)

The most common infection source was urinary (53 patients, 41%), followed by abdominal (34, 26%) and skin and soft tissue (21, 16%); the source was unknown in 18 patients (14%). Two patients had a biliary source, and one had a pulmonary source. The median length of stay was seven days (IQR, 4-14 days), with a mean of 10.0 days and a range of 1-53 days; length-of-stay data were available for 126 of 129 patients (97.7%), with three patients excluded due to absence of a discharge date in the register.

In the exploratory comparison between the two most frequent sources, patients with urinary-source coding-defined sepsis had a substantially higher median age than those with abdominal-source coding-defined sepsis (median, 69 years (IQR, 58-74) vs. 55 years (IQR, 41-67); Mann-Whitney U = 1,296.5, p < 0.001, rank-biserial r = +0.44); three pediatric patients in the abdominal group may marginally contribute to the observed age difference.

In a sensitivity analysis restricted to adults in the full cohort, all 53 urinary-source patients were compared with the 31 adult abdominal-source patients after excluding the three pediatric abdominal cases. Urinary-source patients remained significantly older than abdominal-source patients (median, 69 years (IQR, 58-74) vs. 56 years (IQR, 43.5-68.5); Mann-Whitney U = 1137.5, p = 0.003, rank-biserial r = +0.38). As a second, separate reproducibility check, the analysis was restricted to cases with an explicit ICD-10 source-diagnosis code. This code-confirmed subset comprised 35 urinary-source and 27 abdominal-source patients. Urinary-source patients were again older than abdominal-source patients (median, 69 years (IQR, 57-74) vs. 49 years (IQR, 41.5-65.5); U = 688.0, p = 0.002, rank-biserial r = +0.46). Both analyses supported the same direction of association.

## Discussion

In this retrospective cohort drawn from a complete calendar year at a Romanian county emergency hospital, sepsis predominantly affected older adults and was concentrated within surgical and urological services, with urinary and abdominal infections the leading sources.

The predominance of urinary and abdominal sources, with a comparatively small proportion of pulmonary sepsis, likely reflects this institution's surgical and urological case-mix and referral pattern. Notably, urinary-source coding-defined sepsis was the most common infection category, yet general surgery received more patients than urology; several explanations are possible, including management of complicated urinary infections (such as obstructive uropathy or perinephric collections) by surgical rather than urological teams, local service-allocation practices, or comanagement arrangements not visible in discharge coding; however, the available data do not permit confirmation of any specific explanation. The concentration of cases within general surgery and urology, which together accounted for roughly two-thirds of the cohort, may help inform local prioritization of sepsis-recognition and source-control pathways.

These source patterns can be placed in a wider context. In intensive care cohorts, the respiratory tract typically dominates, as in the worldwide Intensive Care Over Nations audit [8]; in the present cohort, urinary and abdominal sources predominated instead, consistent with a population weighted toward surgical and urological admissions. Urinary tract infection as a notable source has also been reported elsewhere: it ranked third (18.4%) in a Japanese multicentre ICU cohort dominated by respiratory infections [9], was a leading source (~37%) in a United States nationwide analysis [5], and was identified among the principal sites in a tertiary-hospital study from southwestern Romania [10]. These comparisons should be read cautiously given differences in case definition, clinical setting, and study period between cohorts.

In an exploratory comparison, patients with urinary-source coding-defined sepsis had a substantially higher median age than those with abdominal-source coding-defined sepsis (median, 69 years (IQR, 58-74) vs. 55 years (IQR, 41-67); Mann-Whitney U = 1296.5, p < 0.001, rank-biserial r = +0.44), consistent with the known association between urinary sepsis and older age in urological populations; this exploratory comparison was unadjusted and should be regarded as hypothesis-generating.

The demographic profile was also broadly consistent with published data. The male predominance observed here (63.6%) accords with the consistently reported higher incidence of sepsis in men across multiple settings [11,12]. Likewise, the concentration of cases among older adults aligns with the well-described increase in sepsis incidence with age [11,13].

That 129 of 1,147 reviewed infection-related admission records (11.2%) carried a principal sepsis diagnosis. This underscores the frequency with which sepsis was coded at this institution and highlights the prominence of coding-defined sepsis among infectious admissions reviewed. By providing demographic and infection-source data from a Romanian county hospital across a complete year, this study adds to a regional evidence base that remains limited; compared with Western European and North American literature, published hospital-based sepsis data from Romania remain relatively limited and are mainly derived from single-center studies [10], underscoring the value of additional regional descriptions such as this one. The present coding-based approach also parallels a recent large retrospective analysis of 12,089 adult sepsis patients identified by diagnostic coding at a Romanian emergency hospital over an 18-year period [14], reflecting a growing effort to characterize sepsis in Central and Eastern Europe; our findings provide a complementary single-year, county-level perspective from a distinct regional context, and direct comparisons with larger cohorts are limited by differences in study period, institution size, and case-identification methodology.

This study has several limitations. It is retrospective and single-center, which may limit the generalizability of the findings. Case identification relied solely on the principal discharge diagnosis, so patients in whom sepsis was coded as a secondary or comorbid condition under a different principal code (for example, perforated appendicitis, cholangitis, pyelonephritis, or postoperative complication) were not captured; the cohort therefore reflects patients coded with sepsis as the primary reason for admission rather than a clinically adjudicated sepsis population, introducing selection bias and likely undercounting the true burden [7]. The register contained no microbiological data; organisms, blood cultures, and susceptibilities were unavailable, so infection sources were assigned from diagnostic coding and clinical descriptions rather than independent adjudication, and the reported source distribution may reflect classification patterns as much as true epidemiology. No organism-level or antimicrobial-stewardship conclusions can therefore be drawn. The register also lacked illness-severity, hemodynamic, vasopressor, intensive care, comorbidity, and in-hospital outcome data, precluding severity scoring (such as the Sequential Organ Failure Assessment), identification of septic shock, and mortality analysis.

The 11.2% figure represents the proportion of the 1,147 infection-related discharge records that carried a principal diagnosis of sepsis, not the prevalence of sepsis across all hospital admissions. Measured against the full admitted population, coding-defined sepsis accounted for only 0.39% of all ED admissions (129 of 33,250) during the study period, a reminder that the 11.2% reflects sepsis's share of an already infection-selected group, not its true frequency in the hospital as a whole. The discharge service recorded may not capture every team involved in a patient's care, since intensive care involvement, interward transfers, and co-management are not reflected in discharge coding. Administrative coding practices also vary between institutions, which may limit the external comparability of these findings. Cases were not validated against clinical Sepsis-3 criteria; some patients coded as sepsis may not have met organ-dysfunction criteria, and true sepsis cases coded under other principal diagnoses may have been missed. The exploratory age comparison did not account for other factors that could have influenced the observed difference. Data were extracted manually by two reviewers from the printed register, so occasional transcription error cannot be excluded. The joint, rather than independent, classification of records by the two reviewing clinicians, which precluded formal quantification of interrater reliability, is a further limitation affecting the entire case-identification process. Length of stay was right-skewed (median, 7 days; mean, 10.0 days; range, 1-53 days), which should be considered when interpreting the reported mean. Finally, the inclusion of 14 pediatric patients alongside a primarily adult cohort, while intended to provide a complete institutional picture, introduces heterogeneity into combined-cohort summary statistics that should be considered when interpreting figures not stratified by age group.

## Conclusions

Over a complete calendar year at a Romanian county hospital, sepsis predominantly affected older patients and was majority concentrated within general surgery and urology, with urinary and abdominal infections the dominant sources, and a substantial proportion of skin and soft-tissue cases (21 patients, 16.3%).

These descriptive, year-long data provide hospital-based regional information from a setting where such information is limited, and they suggest that local sepsis-recognition and source-control efforts could reasonably prioritize surgical and urological patients and urinary and abdominal sources. Prospective studies capturing microbiological, severity, and outcome data are needed to build on this descriptive foundation.

## Appendices

**Table 3:** STROBE statement - checklist This checklist is based on the STROBE statement for cohort studies. Two items (14c and 15) are marked as not applicable, as this is a retrospective descriptive record review without a follow-up period or in-hospital outcome data, consistent with the limitations stated in the manuscript STROBE: STrengthening the Reporting of OBservational studies in Epidemiology; ICD-10: International Classification of Diseases, Tenth Revision; SD: standard deviation; IQR: interquartile range

Item no.	Section/topic	STROBE recommendation	Location in this manuscript
1a	Title and abstract	Indicate the study's design with a commonly used term in the title or the abstract	Title: "A Retrospective Single-Centre Study"; Abstract states "retrospective, single-centre study"
1b		Provide in the abstract an informative and balanced summary of what was done and what was found	Abstract (structured: Background, Methods, Results, Conclusions)
2	Introduction - Background/rationale	Explain the scientific background and rationale for the investigation being reported	Introduction, paragraphs 1-3
3	Introduction - Objectives	State specific objectives, including any prespecified hypotheses	Introduction, final paragraph
4	Methods - Study design	Present key elements of study design early in the paper	Methods, opening sentence
5	Methods - Setting	Describe the setting, locations, and relevant dates, including periods of recruitment, exposure, follow-up, and data collection	Methods: hospital named; period 1 Jan-31 Dec 2025 stated
6	Methods - Participants	Give the eligibility criteria, and the sources and methods of selection of participants	Methods: category-based filter of the discharge registry followed by individual clinical review; inclusion by principal diagnosis ICD-10 A40/A41; explicit exclusions stated
7	Methods - Variables	Clearly define all outcomes, exposures, predictors, potential confounders, and effect modifiers; give diagnostic criteria if applicable	Methods: age, sex, discharge service, primary infection source, and length of stay all defined; source-category mapping given
8	Methods - Data sources/measurement	For each variable of interest, give sources of data and details of methods of assessment	Methods: Hipocrate OPIS Externari registry described; de-identified variables specified
9	Methods - Bias	Describe any efforts to address potential sources of bias	Methods (joint classification, consensus); Discussion/Limitations (inter-rater reliability, selection bias, coding reliability)
10	Methods - Study size	Explain how the study size was arrived at	Methods: full one-year period used; no formal sample size calculation; all eligible cases included
11	Methods - Quantitative variables	Explain how quantitative variables were handled in the analyses	Methods: continuous variables summarised as mean (SD) or median (IQR)
12a-e	Methods - Statistical methods	Describe all statistical methods, including those used to control for confounding, examine subgroups, handle missing data, address loss to follow-up, and any sensitivity analyses	Methods (Shapiro-Wilk, Mann-Whitney U, two-sided alpha = 0.05); Results (missing sex and length-of-stay data explicitly reported); Results (adults-only sensitivity analysis)
13a-c	Results - Participants	Report numbers of individuals at each stage of the study; give reasons for non-participation; consider use of a flow diagram	Figure 1 (participant flow diagram); Results, opening paragraph
14a-c	Results - Descriptive data	Give characteristics of study participants and information on exposures and potential confounders; indicate number of participants with missing data; summarise follow-up time	Table 1, Table 2; Results (missing sex and length-of-stay data reported). Item 14c (follow-up time) is not applicable: this is a retrospective descriptive record review with no follow-up period.
15	Results - Outcome data	Report numbers of outcome events or summary measures	Not applicable. No in-hospital outcome, severity, or mortality data were available in the source registry; this is explicitly stated as a limitation.
16a-c	Results - Main results	Give unadjusted estimates and, if applicable, confounder-adjusted estimates; report category boundaries when continuous variables were categorized; consider translating relative risk into absolute risk	Results: age, sex, infection source, discharge service, and length of stay reported in full, with age-group categorisation (adult/paediatric) explained
17	Results - Other analyses	Report other analyses done, e.g. subgroup and sensitivity analyses	Results: adults-only sensitivity analysis with independently reassigned, code-confirmed source categories
18	Discussion - Key results	Summarise key results with reference to study objectives	Discussion, opening paragraph; Conclusions
19	Discussion - Limitations	Discuss limitations of the study, taking into account sources of potential bias or imprecision; discuss both direction and magnitude of any potential bias	Discussion, full Limitations paragraph
20	Discussion - Interpretation	Give a cautious overall interpretation considering objectives, limitations, multiplicity of analyses, results from similar studies, and other relevant evidence	Discussion, throughout
21	Discussion -Generalisability	Discuss the generalisability (external validity) of the study results	Discussion (regional and single-centre context); Limitations (retrospective, single-centre design)
22	Other information - Funding	Give the source of funding and the role of the funders for the present study and, if applicable, for the original study on which the present article is based	Disclosures: no financial support was received from any organisation for the submitted work

## References

[1] Singer M, Deutschman CS, Seymour CW (2016). The third international consensus definitions for sepsis and septic shock (Sepsis-3). JAMA.

[2] Rudd KE, Johnson SC, Agesa KM (2020). Global, regional, and national sepsis incidence and mortality, 1990-2017: analysis for the Global Burden of Disease Study. Lancet.

[3] GBD 2021 Global Sepsis Collaborators (2025). Global, regional, and national sepsis incidence and mortality, 1990-2021: a systematic analysis. Lancet Glob Health.

[4] Evans L, Rhodes A, Alhazzani W (2021). Surviving sepsis campaign: international guidelines for management of sepsis and septic shock 2021. Crit Care Med.

[5] Chou EH, Mann S, Hsu TC (2020). Incidence, trends, and outcomes of infection sites among hospitalizations of sepsis: a nationwide study. PLoS One.

[6] von Elm E, Altman DG, Egger M, Pocock SJ, Gøtzsche PC, Vandenbroucke JP (2008). The Strengthening the Reporting of Observational Studies in Epidemiology (STROBE) statement: guidelines for reporting observational studies. J Clin Epidemiol.

[7] Jolley RJ, Sawka KJ, Yergens DW, Quan H, Jetté N, Doig CJ (2015). Validity of administrative data in recording sepsis: a systematic review. Crit Care.

[8] Sakr Y, Jaschinski U, Wittebole X (2018). Sepsis in intensive care unit patients: worldwide data from the intensive care over nations audit. Open Forum Infect Dis.

[9] Abe T, Ogura H, Kushimoto S (2019). Variations in infection sites and mortality rates among patients in intensive care units with severe sepsis and septic shock in Japan. J Intensive Care.

[10] Grigorescu A, Dumitrescu F, Dorobantu S (2025). An Epidemiological Survey of Sepsis in a Tertiary Academic Hospital from Southwestern Romania. Medicina (Kaunas).

[11] Martin GS, Mannino DM, Eaton S, Moss M (2003). The epidemiology of sepsis in the United States from 1979 through 2000. N Engl J Med.

[12] Thompson KJ, Finfer SR, Woodward M, Leong RN, Liu B (2022). Sex differences in sepsis hospitalisations and outcomes in older women and men: a prospective cohort study. J Infect.

[13] Angus DC, Linde-Zwirble WT, Lidicker J, Clermont G, Carcillo J, Pinsky MR (2001). Epidemiology of severe sepsis in the United States: analysis of incidence, outcome, and associated costs of care. Crit Care Med.

[14] Mușat F, Păduraru DN, Bolocan A, Palcău CA, Bunea AA, Ion D, Andronic O (2025). Sepsis burden in a major Romanian emergency center - an 18-year retrospective analysis of mortality and risk factors. Medicina (Kaunas).

